# Comparative pyrolysis kinetics of various biomasses based on model-free and DAEM approaches improved with numerical optimization procedure

**DOI:** 10.1371/journal.pone.0206657

**Published:** 2018-10-31

**Authors:** Miloš Radojević, Bojan Janković, Vladimir Jovanović, Dragoslava Stojiljković, Nebojša Manić

**Affiliations:** 1 University of Belgrade, Faculty of Mechanical Engineering, Fuel and Combustion Laboratory, Belgrade, Serbia; 2 University of Belgrade, Institute of Nuclear Sciences “Vinča”, Department of Physical Chemistry, Belgrade, Serbia; KTH Royal Institute of Technology, SWEDEN

## Abstract

The pyrolysis process of various types of biomass (agricultural and wood by-products) in non-isothermal conditions using simultaneous thermal analyses (STA) was investigated. Devolatilization kinetics was implemented through combined application of model-free methods and DAEM (distributed activation energy model) using Gaussian distribution functions of activation energies. Results obtained were used in the curve prediction of the rate of mass loss against temperature at various heating rates by numerical optimization. The possible calculation of biomass samples behavior under pyrolytic conditions as the summation of their pseudo-components, hemicelluloses, cellulose, and lignin is also explored. The differences between experimental and calculated data are less than 3.20% offering a quality test of applicability of proposed model on the kinetic studies of a wide range of biomass samples. It seems that the most physically realistic model is the decomposition of biomass in three reactions, depending on the composition of the biomass regarding hemicelluloses, cellulose, and lignin. Kinetic model applied here may serve as a starting point to build more complex models capable of describing the thermal behavior of plant materials during thermochemical processing.

## Introduction

Biomass could be described as the result of the storage of sunlight in the form of chemical energy in plants. Through photosynthesis process, sunlight transforms carbon dioxide and water from the atmosphere into a complex of plant polymers for a relatively short period of time [1]. Using these resources for energy production allows the circulation of carbon dioxide, as well as its storage in value-added products. Also, it’s well known that biomass is a fully renewable resource, and its use for production of bioenergy, biofuels, chemicals or other products does not increase the content of carbon dioxide in the atmosphere [2,3]. Therefore, production and utilization of the biomass provide significant benefits to the environment, economy and energy security.

Physical form and distinctive chemical composition of biomass effect on a significant difference compared to fossil fuels and highlight the ecological value [4,5]. The fact that biomass in its composition does not contain (or contains significantly less) sulphur in relation to fossil fuels, gives it an ecological benefit. These valuable effects of using biomass allow substitution of part of coal in combustion systems or co-combustion biomass and coal in large power plants [6–8]. Cellulose and hemicellulose as two of the three main components of the most biomass feedstock are sugar polymers, which benefits could be utilized by different conversion process [9]. These components can be used for pyrolysis, gasification, torrefaction, fermentation or other thermochemical processes for obtaining valuable fuels, chemicals, and different materials or can be combusted for the production of heat, steam, and electricity [10–12].

Although, some properties of biomass such as small bulk density, high moisture content and non-uniform chemical composition could make complicate procedures for collection, transportation, and storage as well as for utilization process of biomass [13]. These issues could be also occurred for the same type of biomass feedstock but collected from different regions [14]. Regarding the aforementioned, it is extremely important to perform a detailed characterization of different biomass feedstock from a specific region in order to obtain the valuable data for the efficient utilization by specific thermal conversion process.

In this study, the pyrolysis kinetics of various biomass samples such as: corn brakes, sawdust, chemically treated (MDF) sawdust, wheat straw and hazelnut shells were examined. Understanding the kinetics of pyrolysis is vital to design, optimize, and scale up the industrial biomass conversion applications. The selected biomasses were used to evaluate their untapped thermal potential. Despite the wide application of biomass in the energy sector, there are still some complications which prevail due to the chemical and physical attributes, such as low thermal efficiency as it is highly fibrous, channeling and bridging, and transportation costs. Torrefaction and pelletization could be useful to solving these problems to some extent. But before proceeding to use a different kind of biomass, it is very essential to have comprehensive knowledge about its chemical and physical behavior as well as the kinetics of the thermo-chemical process of the biomass.

The objective of this study is to evaluate the kinetics parameters for thermal decomposition of various biomasses with the help of thermal analysis (TA) techniques equipped for simultaneous measurements. The indicated experimental techniques are useful tools in determination of kinetic parameters of reactions occurring in the solid state. A considerable number of kinetic methods capable of quantitatively characterizing these reactions have been developed over the years and are generally categorized as model-fitting and model-free methods [15]. In this study, we have chosen to use the differential isoconversional analysis, Friedman’s (FR) method, as well as the integral isoconversional analysis method, such as Kissinger-Akahira-Sunose (KAS). These methods were used to determine the activation energy (*E*) dependency as a function of the conversion degree (α), without any previously knowledge of the reaction model [15]. The analysis of *E* dependency using these methods has been proven to be helpful in exploring the mechanisms and prediction of kinetics for biomass pyrolysis. The interpretation of the dependence of activation energy values upon the conversion may provide useful mechanistic clues, such as the number of presumable reaction steps.

For example, it has been demonstrated that the use of Friedman’s (FR) method applied to deconvoluate thermogravimetric signals of three pseudo-components (cellulose, hemicelluloses and lignin) obtained from the whole experimental signal of the raw material could satisfactorily predict the kinetic rate of the same plant material [16]. The same authors observed that the resulting activation energy for these pseudo-components was consistent with values reported for model compounds ascribed as hemicelluloses, cellulose, and lignin. The pyrolysis of the raw material was modeled with good approximation by three independent reactions, whose kinetic parameters were determined using model-free methods. So, the *E* values can serve as the *reactivity criterion* for biomass pyrolysis. However, the numerous attempts in modeling of pyrolysis kinetics based on global reaction models or the individual contribution of model compounds have been made and often failed at describing the entire decomposition process of plant biomass. The reason lies in the fact that different biomass materials show different behavior because of their inherent physico-chemical characteristics, which can lead a criterion on the mathematical description selected for its modeling. Therefore, the chemical and physical differences of the reactive species lead to application of DAEM (distributed activation energy model), as Burnham and Braun outlined in a detailed review in 1999 [17]. In this sense, DAEM is considered as an accurate and versatile approach to model the pyrolysis process. In the last years, the model has been thoroughly employed to analyze the kinetics of pyrolysis of different biomass feedstocks [18–21]. This model assumes that the distribution of reactivity caused by the reaction complexity can be represented by a set of independent, parallel reactions. Reactivity distribution of these reactions, described by a distribution of pre-exponential (frequency) factors and the *E* values, can be solved mathematically. Specific mathematical forms appearing in the literature are the Gaussian, Weibull and Gamma distributions [17]. In most cases, the DAEM introduces an infinite number of reactions following a given distribution of the *E*_*a*_ values and constant pre-exponential factors (*A*) are assumed. In the present work, the continuous DAEM coupled with model-free (isoconversional) approach are employed to perform a systematic investigation of the pyrolysis characteristics of various biomass feedstocks. This approach allows detection, whether there is a complex interaction phenomenon among constituents of the particular biomass sample. The current work allows comparison of different methods based on simultaneous thermal analyses (STA) for kinetic parameters determination and accurate prediction of pyrolysis process related to various classes of biomass.

## Materials and methods

### 2.1. Used materials and sampling procedure

For simultaneous thermal analyses (STA), five different biomass samples were used. Three samples among them are different types of agricultural biomass samples: corn brakes, wheat straw and hazelnut shells. The remaining two samples represent the wood processed products: sawdust and factory-formed wood product as chemically treated (MDF—medium density fibreboard) sawdust. The selected samples have good propositions for the use in the pyrolysis pretreatment for further exploitation in, for example, the entrained-flow gasification process. For actual process, the biomass needs to be pretreated to significantly increase its heating value and to make it more readily transportable. Such pretreatment is carried out through a pyrolysis, where the effects of pyrolysis temperature on the yield, composition and heating value of the gaseous, liquid and solid products can be extremely important. The biomass decomposition kinetics is important because determines the composition of end-products, and thus, the utilization options for the observed product as well as, influences the technical parameters of pyrolysis process. This last has significant effects on the economic feasibility of biomass pyrolysis.

Corn was grown and collected on domestic fields in central region of the Republic of Serbia near town Jagodina at location: Latitude 43.998062, Longitude 21.275547, N43°59'53.0" E21°16'32.0". After peeling, corn brakes were left on a pile as industry leftovers for 2 months. Hazelnut shells came from suburban region of Serbia, near Capital city, from village Begaljica at location: Latitude 44.616032, Longitude 20.645596, N44°36'57.7" E20°38'44.2". After removing hazelnuts’ inner part, shells were collected in bags and left for 8 months before they were brought to the laboratory. Wheat straw was brought from the Southern Vojvodina near Banatski Brestovac at location: Latitude 44.745227, Longitude 20.795401, N 44°44'42.8" E 20°47'43.4" and it was already dried before sample preparation started. Sawdust was collected from the process of cutting beech log wood in wood processing industry near Lapovo at location: Latitude 44.167978, Longitude 21.103702, N44°10'04.7" E21°06'13.3", while the chemically treated sawdust was obtained from local carpenter workshop in Belgrade at Milana Rakica street at location: Latitude 44.796917, Longitude 20.503607, N44°47'48.9" E20°30'13.0".

According to standard EN ISO 14780 [22] for solid biomass fuel sample preparation each biomass sample was prepared for experimental tests. Sample was removed from the transportation packing, and then it is placed inside the oven at 378 K for 2 h, in order to define moisture left on the inner surfaces of the packing. This amount of moisture was included in the calculation of the total moisture content in the sample according to standard EN ISO 18134 [23]. Further, regarding to pre-drying process of the sample, which is necessary to remove the residual moisture during preparation, sample was placed on a plate and left to reach moisture equilibrium with laboratory atmosphere conditions for 24 h. After that period of time, sample was reduced in two steps using cutting mill, after which it was sieved through the 1 mm (18 Mesh sizes) sieve.

Finally, the obtained undersize was declared as a general analysis sample and divided into 3 test portions for further testing. First portion of prepared sample was used for determination of ultimate analysis (carbon, hydrogen and nitrogen content), according to standard EN ISO 16948 [24]. The results of proximate analysis (total moisture, ash, volatile matter and char content) were obtained from the second portion of the prepared sample. This analysis was performed according to standard procedures EN ISO 17225–1 [25]. The higher heating value (HHV) and lower heating value (LHV) for tested samples were determined using calorimeter laboratory equipment with an oxygen bomb (IKA C200), according to the standard EN ISO 18125 [26].

### 2.2. STA measurements

The STA was performed on the third portion of the prepared biomass sample, and all of the obtained results were used for studying the pyrolytic kinetics.

NETZSCH STA 445 F5 Jupiter system was used for STA experimental tests for all examined samples. All STA measurements were performed between March 20^th^ and April 5^th^ 2017. Inert atmosphere was provided to maintain the pyrolysis process using high purity nitrogen (Class 4.6) as a carrier gas. At the same time, nitrogen was used as a protective gas in order to keep the high sensitive internal balance (0.1 μg). Both carrier and protective gas flow were set to *φ* = 50 mL min^-1^. The weight measurements were carried out using internal balance which provided the following results: corn brakes– 15.45 ± 0.10 mg, hazelnut shell– 29.35 ± 0.10 mg, wheat straw– 10.80 ± 0.50 mg, sawdust– 20.70 ± 0.50 mg, and chemically treated sawdust– 13.70 ± 0.50 mg, respectively. Crucibles that were used for tests are made of alumina, and during each measurement they were filled approximately up to the half. Crucible’s lid was placed on the top, so the optimum heat transfer could be realized. Each sample was tested using three different heating rates, one for each measurement. It was considered that heating rates of 5, 10 and 20 K min^-1^ are the best for kinetic parameters comparison. Using these heating rates, the samples were heated from room temperature up to 873 K. During all measurements, the sample temperature controller (STC) was turned off, so the set temperature (873 K) is referred to the furnace temperature. Temperatures presented on diagrams are the sample temperatures that, due to the construction of the furnace, never reached the set temperature. This fact resulted in better temperature curve linearity, than it would be if STC was turned on. The STA 445 F5 Jupiter runs under the versatile software and includes all operating tools to obtain a reliable measurement and evaluate the resulting data, or even carry out complicated analyses.

## Theoretical background

Pyrolysis is generally a complex process and it is difficult to discover kinetic models which explain the mechanism of thermal decomposition. A full kinetic analysis of complex systems is generally not feasible, but some kind of “effective” or “average” kinetic description is still needed. In many kinetic formulations of the solid state reactions, it has been assumed that isothermal homogeneous gas or the liquid phase kinetic equation can be applied [27].

The overall rate of reactions is commonly described by the following equation:

$$\beta ∙(\frac{d\alpha }{dT})\equiv \frac{d\alpha }{dt}=k(T)∙f(\alpha )=A∙exp(-\frac{E}{RT})∙\;f(\alpha )$$ (1)

where *β* = d*T*/d*t* is the heating rate, *k*(*T*) = *A*·exp(–*E*/*RT*) is the temperature-dependent rate constant expressed by the Arrhenius law, α is the normalized conversion of the raw materials decomposition, defined as: α = (*m*_o_ − *m*_*T*_) / (*m*_o_ − *m*_*f*_) where *m*_o_ is the initial mass of the sample, *m*_*T*_ is the mass of the sample at temperature *T*, and *m*_*f*_ is the mass of the sample at infinite time as a saturation value; All of the masses are on dry basis. *f*(α) depends on the mechanism of the thermal decomposition, i.e. the reaction mechanism function, *E* is the activation energy, while *A* represents the pre-exponential factor, *R* is the gas constant, and *T* is the absolute temperature.

Model-free methods provide effective activation energy and they have the advantage of allowing the determination of the activation energy and the pre-exponential factor without the need of anticipating the reaction mechanism. The most popular model-free (isoconversional) kinetic methods are the Friedman (FR) [28] as differential isoconversional method and Kissinger-Akahira-Sunose (KAS) [29,30] as integral isoconversional method.

### 3.1. Friedman method

The Friedman (FR) method [28] is based on the Eq (1), relates to the logarithm of the reaction rate to the inverse temperature at a given constant conversion value and the heating rate, as follows:

$${ln(\frac{d\alpha }{dt})}_{\alpha ,i}=ln[A∙f(\alpha )]-\frac{E}{RT_{\alpha ,i}}$$ (2)

From this equation, the first right-side member is constant, at a given heating rate (*β*_*i*_) and conversion value (*α*). Thus, the correspondent plot gives the straight lines with a slope that is directly proportional to the activation energy (*E*) and, therefore, it can be derived from there.

### 3.2. Kissinger-Akahira-Sunose method

The Kissinger-Akahira-Sunose (KAS) [29,30] method is based on the Coats-Redfern approximation [31] of the temperature integral, *p*(*x*) ≈ exp(-*x*)/*x*^2^, and can be applied without any assumption concerning kinetic model, and the KAS relationship can be expressed as:

$$ln(\frac{\beta }{T^{2}})=ln[\frac{AR}{Eg(\alpha )}]-\frac{E}{RT}$$ (3)

Activation energy values at various α’s can be obtained from the set of the slopes of the straight lines of ln(*β*/*T*^2^) against 1/*T*. If the reaction follows a single-step mechanism, the respective *E* values are expected to be similar. This means that it should not change considerably with conversions. A great change in the magnitude of these values with a change of α indicates the occurrence of a multi-step reaction(s) that definitely do not fit the single-step reaction mechanism.

### 3.3. DAEM approach

Based on prior studies on the kinetics of coal devolatilization [32], we expect that it is probably more realistic to assume that biomass devolatilization kinetics is also likely to involve distributed activation energies. Considering this possibility, we were motivated to see how use of this model can affects the expected biomass pyrolysis performances. The DAEM, in which the activation energy of every biomass decomposition reaction distributes over a range, provide a way to account for the inherent non-uniformity of chemical bonds in biomass. In this study, the DAEM kinetics was applied in the assessment how it affects on the resulting products restricted in the case, where the fixed size particles were involved.

In contrast to conventional Arrhenius kinetics, DAEM assumes that activation energies are distributed over a range, and thus they are described in terms of probability density function, *f*(*E*). DAEM assumes the existence of distribution of reactivity which results from the reaction complexity as represented with enough reliability by a series of independent, parallel reactions, which are characterized by their own activation energy and the pre-exponential factor. It is commonly accepted as a good approximation to assume a pre-exponential factor one for all reactions. The final calculation equations related to conversion curves and pyrolysis rate curves used in this work can be found elsewhere [33,34].

The complexity of lignocellulosic biomass pyrolysis is such that a continuous distribution *f*(*E*) of the activation energies is assumed where $\int_{E}^{E+\Delta E}f\left(E\right)dE$ describes the probability that chemical groups with a sample have an activation energy in this range *E* and *E* + Δ*E*. The conversion fraction of potential volatile material with activation energies between *E* and *E* + *dE*, *d*α, was given in a form:

dα=f(E)dE (4)

where the initial distribution curve can be obtained from dependency α = α(*E*) through differentiation *d*α/d*E* ≡ *f*(*E*). It can be seen from above description that *f*(*E*) is the key feature in DAEM. In application of DAEM, the distribution curve usually takes the Gaussian (Normal) form such as:

$$f(E_{\alpha })=\frac{1}{\sigma {\left(2\pi \right)}^{1/2}}exp[-\frac{{\left(E_{\alpha }-\mu \right)}^{2}}{2\sigma^{2}}]$$ (5)

where *E*_α_ is the activation energy (kJ mol^-1^) values estimated by Friedman’s isoconversional method, *σ* is the standard deviation or width of the distribution curve (kJ mol^-1^), and *μ* is the mean activation energy (kJ mol^-1^). Most laboratory experiments involving pyrolysis of lignocellulosic biomass which are performed at temperatures linearly varying with time (*t*) from a starting temperature *T*_o_. Under the linear heating program, *T*(*t*) = *T*_o_ + *β*·*t* (*β* is the heating rate (K min^-1^)), the general *n*-th order DAEM equation with the Normal (*μ*, *σ*) distribution *f*(*E*) curve of activation energies can be derived [33].

In the case where pyrolysis process of lignocellulosic material can be described with multi-step kinetics, the composite distribution function of activation energies *f*(*E*), can be presented as:

$$f(E)=w∙f_{1}(E)+w∙f_{2}(E)+(1-w)∙f_{3}(E)$$ (6)

where *f*_1_(*E*), *f*_2_(*E*) and *f*_3_(*E*) are the Gaussian distributions with distribution parameters *σ*_1_, *μ*_1_, *σ*_2_, *μ*_2_, and *σ*_3_, *μ*_3_, corresponding to the decomposition processes of individual biomass pseudo-component, respectively; *w* is the weight parameter. Consequently, the *f*(*E*) represents the complex, generic distribution curve of various activation energy values.

The parameter estimation can be performed by using a global optimization technique—Pattern Search Method (PSM), which is derivative-free and direct search method, which is superior to the other direct search methods (Simplex Method, Grid Search Method, or Simulated Annealing Optimization Method). The unknown parameters can be determined from the objective function (*OF*) expressed through pyrolysis rate curves as:

$$OF=\frac{{\left\{\frac{\sum_{0}^{N_{k}}{\left[{\left(\frac{d\alpha }{dt}\right)}_{N_{k}}^{exp}-{\left(\frac{d\alpha }{dt}\right)}_{N_{k}}^{calc}\right]}^{2}}{N_{k}}\right\}}^{\frac{1}{2}}}{{\left(\frac{d\alpha }{dt}\right)}_{max}^{exp}}\times 100$$ (7)

where *N*_*k*_ is the amount of the data points, (dα/d*t*)_*Nk*_^*exp*^ is the experimentally observed value measurement, (dα/d*t*)_*Nk*_^*calc*^ is the calculated value obtained by numerical solution of differential form of the rate-law equation [33], for a given set of parameters, while (dα/d*t*)_*max*_^*exp*^ is the maximum experimental value. There are many optimization routines that are used to determine which combination of parameter values results in the minimum *OF* value, and this is currently an active field of research.

## Results and discussion

### 4.1. Proximate and ultimate analyses

Table 1 shows the results of proximate and ultimate analyses of all tested biomass (corn brakes, wheat straw, hazelnut shell, sawdust, and MDF sawdust, respectively) samples.

**Table 1:** Proximate and ultimate results for the various tested biomass sample.

**Corn brakes**			
**Proximate analysis (wt %)**		**Ultimate analysis**^b^ **(wt %)**	
Moisture	8.58	C	47.97
Volatile matter	73.94	H	6.87
Fixed carbon	16.00	O^c^	42.89
Ash	1.48	N	0.66
HHV (MJ kg^-1^)	16.72	S	0.00
LHV^a^ (MJ kg^-1^)	15.10		
**Wheat straw**			
**Proximate analysis (wt %)**		**Ultimate analysis**^b^ **(wt %)**	
Moisture	11.63	C	44.12
Volatile matter	65.32	H	6.34
Fixed carbon	15.17	O^c^	39.99
Ash	7.88	N	0.63
HHV (MJ kg^-1^)	15.29	S	0.00
LHV^a^ (MJ kg^-1^)	13.91		
**Hazelnut shell**			
**Proximate analysis (wt %)**		**Ultimate analysis**^b^ **(wt %)**	
Moisture	9.27	C	50.12
Volatile matter	68.08	H	6.66
Fixed carbon	20.97	O^c^	40.53
Ash	1.67	N	0.86
HHV (MJ kg^-1^)	18.20	S	0.00
LHV^a^ (MJ kg^-1^)	16.68		
**Sawdust (*Beech*)**			
**Proximate analysis (wt %)**		**Ultimate analysis**^b^ **(wt %)**	
Moisture	7.16	C	49.46
Volatile matter	74.14	H	6.82
Fixed carbon	17.02	O^c^	41.88
Ash	1.68	N	0.03
HHV (MJ kg^-1^)	17.78	S	0.00
LHV^a^ (MJ kg^-1^)	16.21		
**Chemically treated sawdust (*Beech*)**			
**Proximate analysis (wt %)**		**Ultimate analysis**^b^ **(wt %)**	
Moisture	5.40	C	48.63
Volatile matter	79.62	H	6.55
Fixed carbon	14.40	O^c^	42.20
Ash	0.58	N	2.01
HHV (MJ kg^-1^)	18.15	S	0.00
LHV^a^ (MJ kg^-1^)	16.62		

^a^ Calculated according to [19].

^b^ On a dry basis.

^c^ By the difference.

It can be seen from Table 1 that all biomass samples are characterized by high volatiles matter, ranging from 65.32–79.62%, which makes them desirable for a good regulation of combustion or gasification processes. Among tested samples, wheat straw shows the highest ash content value (7.88%), because of the fact that among all agricultural by-products, the high ash contents of straws [35] interfere with the pulping process (apropos water must be spray onto the wheat straw when cutting it in pulp mill to prevent raising of the dust), the chemical recovery of pulping chemicals and the utilization of straws as fuels. The raw materials have moisture content in a range 5.40–11.63% which can be attributed to the open air conditions as well as to wet separation process. In addition, it can be observed from Table 1 that chemically treated (MDF) sawdust (*Beech*) is characterized by the highest value of volatile matter in respect to other samples, which means it is characterized by the highest conversion, in comparison to biomass sample with the highest fixed carbon (hazelnut shell, Table 1). From a theoretical point of view, corn brakes, sawdust and MDF sawdust can be suitable for good production of bio-oil, while the hazelnut shell would be the best biomass sample for production of bio-char (Table 1). All tested samples have the HHV values ranging from 15.29–18.20%, which belongs to the heating values of biomass derived bio-chars (11.83–44.20%) [36,37]. Generally, the biomasses with high HHV values make them very attractive for the source-feeds for clean energy production instead of fossil-based solid fuels (for example, hazelnut shells stand out; Table 1).

In addition, the concentration of C and H is the lowest for wheat straw, while their highest concentration was identified for hazelnut shell and sawdust (Table 1). Almost negligible content of N has been identified for all biomass samples, except for MDF sawdust (Table 1), which means that the potential toxic NO_x_ emissions during the studied process are almost eliminated. The wheat straw has a something lower content of O compared to other samples, which may indicate on the creation of smaller amounts of inorganic vapors in combustion processing. However, all samples show the presence of oxygen more than 30% which means that they can be essential in deoxygenation. The agricultural biomass has a low heating value due to its high ash content, where it was reported [38] that biomass with a high ash content is not an ideal fuel source, because the ash content is one of the main factors that directly influence the heating value of biomass. High carbon and low oxygen in biomass as compared to coal (73.1% of carbon and 8.7% of oxygen) are favorable for combustion applications, while the higher proportion of carbon (relative to hydrogen and oxygen content) (for example, wheat straw and hazelnut shells in Table 1) increases the energy content of a fuel because energy contained in carbon-carbon bonds is greater than that of carbon-oxygen and carbon-hydrogen bonds [39].

### 4.2. TGA-DTG curves of pyrolysis processes of various biomass samples

**Fig 1:**
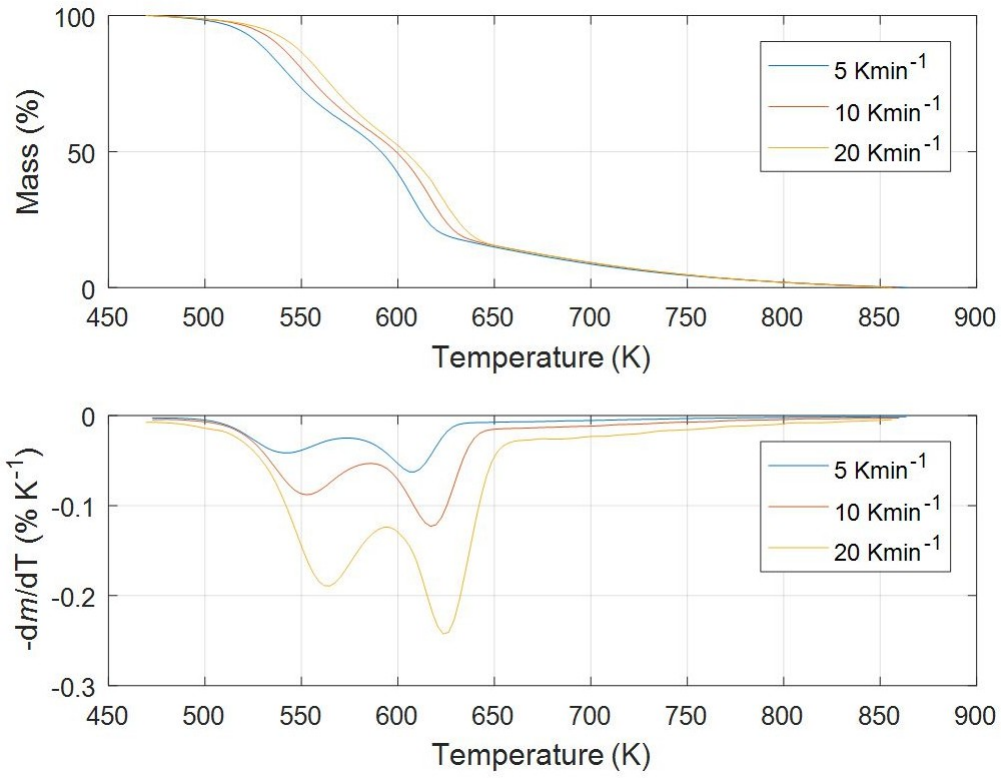
TG-DTG curves of pyrolysis process of corn brakes at different heating rates in a nitrogen atmosphere.

**Fig 2:**
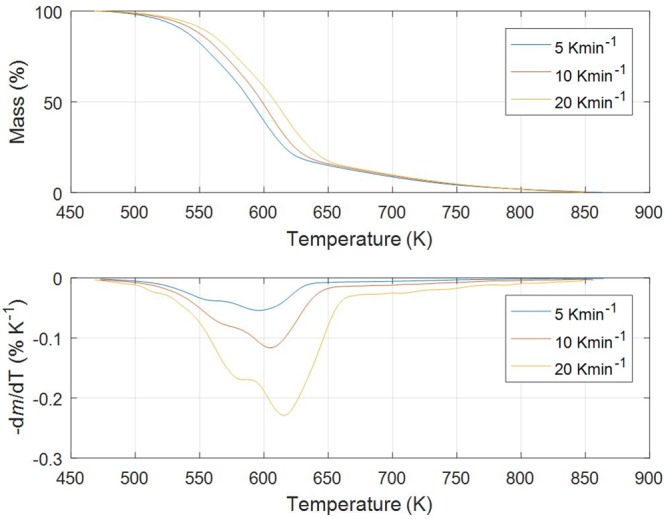
TG-DTG curves of pyrolysis process of wheat straw at different heating rates in a nitrogen atmosphere.

**Fig 3:**
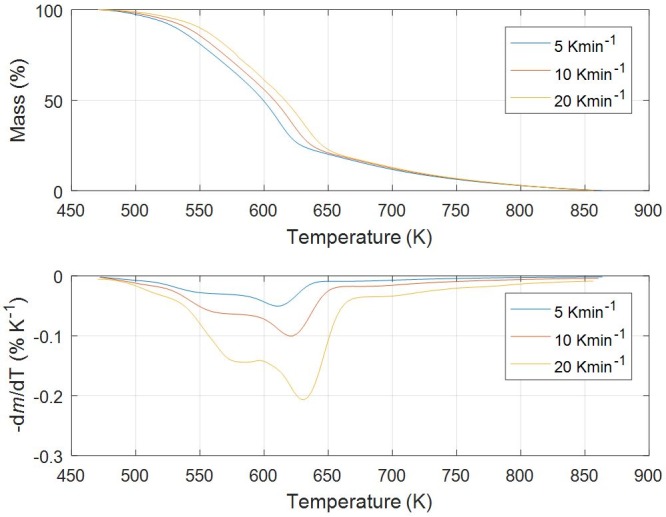
TG-DTG curves of pyrolysis process of hazelnut shell at different heating rates in a nitrogen atmosphere.

**Fig 4:**
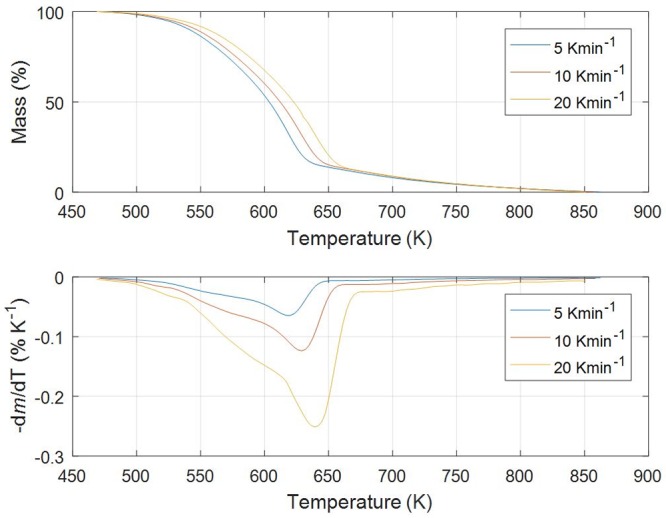
TG-DTG curves of pyrolysis process of sawdust (*Beech*) at different heating rates in a nitrogen atmosphere.

**Fig 5:**
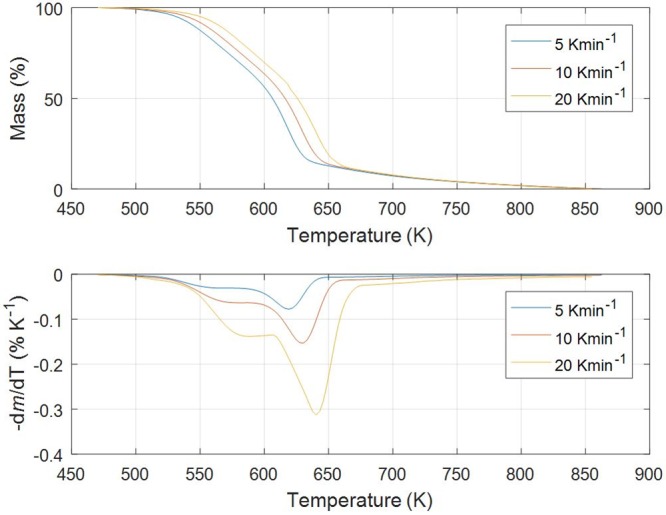
TG-DTG curves of pyrolysis process of chemically treated sawdust (*Beech*) at different heating rates in a nitrogen atmosphere.

Figs 1–5 show experimental TG (Thermogravimetric)–DTG (Derivative thermogravimetric) curves for thermal decomposition of corn brakes, wheat straw, hazelnut shell, sawdust and MDF sawdust under an atmosphere of nitrogen at the heating rates of 5, 10 and 20 K min^-1^, respectively. Consistent with Figs 1–5, the moisture was removed from raw materials up to 445 K, and main decompositions of investigated samples started around 450 K and finished around 900 K.

For pyrolysis of corn brakes, the TG-DTG curves (Fig 1) show three distinct mass loss stages could be determined (Δ*T*_I_ = 470–580 K, Δ*T*_II_ = 580–650 K, and Δ*T*_III_ > 650 K, respectively), and it is in agreement with researches related to corn by-products [40]. The first stage corresponds to decomposition process of hemicelluloses, with most decomposition taking place in above-indicated temperature region (Δ*T*_I_). In the second stage (second decomposition), the decomposition of cellulose occurs, indicated by Δ*T*_II_, with very less solid residue remaining after 680 K. Among the three pseudo-components of biomass, lignin is the most difficult one to decompose. Its decomposition occurs slowly under a wide range of temperature from the ambient up to 850 K, including stage designated by the temperature region, Δ*T*_III_. In a third stage (> 650 K), the char-forming reactions take place, where also the highest solid residue is left after pyrolysis of lignin. However, some reported that pyrolysis of heavier volatiles such as lignin occurs from temperature as low as 423 K up to 1173 K, since it is more thermally stable in contrast to cellulose and hemicelluloses [41]. Hemicelluloses, cellulose, and lignin are the major pseudo-components of biomass samples and decomposition of those ingredients occurred in indicated temperature ranges [40].

In the case of pyrolysis processes of wheat straw and hazelnut shell (Figs 2 and 3), TG-DTG curves show very similar behavior to thermal analysis curves related corn brakes pyrolysis (in relation to indicated temperature intervals, where pyrolytic reactions occur), but it was identified certain “disturbing shape” behavior in a DTG peak related to hemicelluloses decomposition. Namely, with an increasing of heating rate (in trend of temperature rise), the well-defined peak present in corn brakes pyrolysis (Fig 1) is transformed into the “shoulder”, which is particularly prominent in the case of hazelnut shell pyrolysis (Fig 3). In contrast, for sawdust (*Beech*) pyrolysis (Fig 4), the shape of the existing “shoulder” goes into a single peak, which is previously expressed in clear two-braking lines DTG feature (in a temperature interval from 470 K up to 610 K) at all heating rates (Fig 4). Furthermore, for MDF sawdust pyrolysis (Fig 5), the second DTG peak is very pronounced (especially at the highest heating rate), but with the appearance of a conditional “shoulder” related to the first decomposition process, which was not present in sawdust pyrolysis. These changes can obviously be seen from the shapes of TGA curves at all heating rates, for all tested biomasses (Figs 1–5). Due to the large extent of mass loss occurring during the first two decomposition processes, this is known as the active pyrolysis region. This region is characterized by significant drop in mass of the samples due to the liberation of volatile hydrocarbon from rapid thermal decomposition of hemicelluloses, cellulose, and some part of the lignin. The final stage involves endothermic decomposition of lignin at temperatures above 670 K [42], denoted by the long tailing in DTG curves of biomass samples (Figs 1–5). Also, the residue masses from TG experiments agree very well with the proximate analysis of tested biomasses (Table 1). In addition, it can be observed that an increase in heating rate shifted the thermal evolution profiles (TG curves) of various biomass samples to higher temperatures. This effect is known as thermal hysteresis or thermal lag. That was because higher heat transfer efficiency would be achieved at the lower heating rate. Nevertheless, an increase in heating rate would generate a larger temperature gradient across the poor heat conductor of biomass particle, and ultimately facilitate the decomposition rate [42]. At the high heating rates, separate DTG peaks did not arise because some of them were decomposed simultaneously and several adjacent peaks were united to form overlapped boarder and higher peaks. This is consequence of heat and mass transfer limitations. In this respect, with an increase in heating rate, the temperature in furnace space can be a little higher as the temperature of a particle and the rate of decomposition are higher than the release of volatiles.

From above results, the differences in the molecular structures and chemical nature of three pseudo-components account for dissimilar observed behaviors. TG patterns of lignocellulosic biomass can be stated as convolution of TG curves of three main pseudo-components, with extent of convolution depending on percentage composition of individual components. Namely, it is obvious that the variations in appearance in DTG curves are primary related to the variation in percentage of hemicelluloses presented in current biomass samples. Exothermic effects can be identified in decomposition patterns of hemicelluloses and lignin that are related to their high charring nature, and which is in contrast with decomposition of cellulose taking place by the full volatilization [43].

### 4.3. Model-free studies

**Fig 6:**
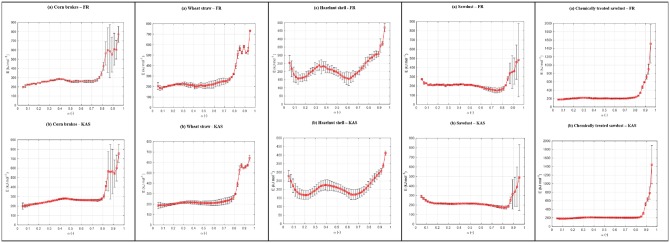
The dependencies of *E* = *E*(α) for pyrolysis process of various biomasses.

Based on model-free approaches, the *E* = *E*(α) dependencies for pyrolysis of tested biomasses were estimated. Fig 6 shows isoconversional dependencies of activation energy values in a conversion range α = 0.05–0.95 with an indicated 95% confidence interval (with displayed errors of calculated *E* values).

It can be observed from Fig 6 that for all biomass samples, the activation energy trends are similar considering both methods (FR and KAS). The small differences among the activation energy values determined by means of two isoconversional methods may be caused by using a different calculation method, or experimental data processing method (FR method does not use any approximation, which is the opposite in the case of KAS method). Table 2 lists the mean values of activation energies estimated by FR and KAS methods, for all analyzed biomass samples.

**Table 2:** The mean values of activation energies determined by FR and KAS methods for investigated pyrolysis processes in considered conversion range (α = 0.05–0.95).

Sample	Corn brakes	Wheat straw	Hazelnut shell	Sawdust (*Beech*)	Chemically treated (MDF) sawdust
**FR**					
*E*_*mean*_ (kJ mol^-1^)	316.38	279.44	222.35	225.04	278.02
**KAS**					
*E*_*mean*_ (kJ mol^-1^)	300.57	265.06	216.35	227.76	260.73

Considering FR method (also, similar to the KAS), based on obtained *E*_*mean*_ values, the following order of pyrolysis reactivity (from the fastest (“facilitated”) to the slowest (“complicated”)) can be established, as: Hazelnut shell (HS) < Sawdust (SD) < Chemically treated (MDF) sawdust (CTSD) < Wheat straw (WS) < Corn brakes (CB). The various pyrolysis reactivity can arises from different lignocellulosic content presents in actual biomasses, as well as on the processing parameters such as reaction temperature range, effect of particle sizes, the heating rate impacts, etc. In addition, secondary intra-particle reactions may play a significant role in exothermic behavior of the process and thermal runaway, which can also have an impact on the type of kinetic mechanism and therefore on the activation energy magnitudes.

The shapes of *E* ― α dependency for CB and WS are very similar, where three zones can be distinguished: the low and medium conversions on one side (up to α = 0.70), and high conversion in the other side (over α = 0.70). The HS exhibits a different shape of *E* ― α dependency from the rest of biomasses, but with a much greater magnitude of variance of the *E* values throughout the entire pyrolysis process (Fig 6). Finally, SD and CTSD in comparison with other types of biomass show a different trend of the change of *E* values with α, but also a mutual, different form of *E* ― α dependencies (Fig 6). In the case of CTSD, there is no significant variation in *E* with α up to α = 0.80, but above this value there is a rapid increase in the *E* value. For sawdust (SD) pyrolysis, the *E* exhibits increased instability through its variation over three reaction zones (Fig 6). From presented results in Fig 6, at lower conversions, the initial energy for water evaporation is different for various biomasses.

This observation indicates that the physical water, ascribed as the moisture content (Table 1) and generally residing outside the cell walls, should have a limited impact on the chemistry of slow pyrolysis process and, therefore, on the associated levels of activation energies.

Considering the *E* values calculated for pure cellulose (C), hemicelluloses (H) and lignin (L) reported in literature (C: 183.81 kJ mol^-1^, H: 108.65 kJ mol^-1^, and L: 226.24 kJ mol^-1^) [44], the estimated activation energies for all observed biomasses give higher values than those for isolated pseudo-components in corresponding temperature ranges (intended for their decompositions and described by the behavior of DTG curves (Figs 1–5)). In all cases, in the range of 0.10 < α < 0.35(0.40) we may expect a hemicelluloses decomposition with average value of *E* ~ 200 kJ mol^-1^ (except for CB and HS). On the other hand, the cellulose decomposition occurs in a middle conversion range approximately 0.40 < α < 0.80 (except for HS), with average value of *E* ~ 240 kJ mol^-1^ which is generally a slightly higher than value predicted for cellulose decomposition in accordance with Broido-Shafizadeh model [45]. Namely, this model suggests that cellulose transforms into active-cellulose first (the E value is around 270 kJ mol^-1^), and then that the active-cellulose decomposes into volatiles and char (the *E* values for char and volatiles are all around 200 kJ mol^-1^) [46]. Taking into account the above-established value of 240 kJ mol^-1^ which lies between these transitional *E* values, it can be concluded that the decomposition of cellulose for given samples (except for HS due to the large variation in *E*’s) is in good agreement with Broido-Shafizadeh model. The lignin decomposition occurs at higher conversions (predominantly above α = 0.75/0.80) (certain deviation occurs for HS) (Fig 6), with very high *E* values. This trend follows the simultaneous increasing trends in conversion and temperature values. Namely, this suggests that the decomposition of lignin involves numerous reactions with different activation energies. This behavior is representative in the sense that the lignin abounds of the great variety of bonds, and also of the multi-phasic character of its conversion. The last phenomenon can be explained by an increase in the thermal stability as a result of the increasing aromatic character of the lignin-derived char when higher temperatures are reached, turning lignin into a highly cross-linked carbonaceous material. However, a great dissipation in *E* values for lignin decomposition considering all observed biomasses (Fig 6), may arises from alterations of the chemical structure of lignin in these samples (percentage representation of guaiacyl (G) or syringyl (S) subunits). In addition, the different pyrolysis behavior of HS (Fig 6) comparing other biomasses, can be explained by with increased interaction between cellulose and lignin, which can create a reactive complex and cause disturbing *E* ― α dependency in comparison with other samples. This interaction may probable occurs in an active pyrolysis zone. The formation of cellulose-lignin complex can be formed from oligomers and intermediates from cellulose and lignin through hydrogen bonds, and thus increase the biomass reactivity. In mentioned reactivity zone, there are many primary products from cellulose and lignin formed by the breakage of weak bonds. Both cellulose and lignin have numbers of ―OH groups, so the formation of cellulose-lignin complexes is possible. However, this formation is not excluded in the case of other samples, but this process is much less pronounced, which depends to a large extent on the content ratio of cellulose and lignin in these samples. Generally, considering all isoconversional results which are shown in Fig 6, it can be concluded that model-free approach is more suitable for describing the kinetics of biomass which exhibits only one peak in the DTG curves, which is not the case with our tested biomass samples (Figs 1–5). The use of the model-free model alone cannot enable a detailed description of pyrolysis mechanism, because of the individually complex and imposed cellulose, hemicelluloses and lignin kinetics. Their kinetics is characterized by a large number of reactions with specific values of activation energies distributed in certain ranges, forming a distribution that characterizes each pseudo-component. Therefore, a different and more sophisticated approach is needed, which would use the information obtained from model-free models in order to well describe the reaction curves of the pyrolytic process. Therefore, it is necessary to optimize the kinetic parameters of pyrolysis processes. This optimization includes separate features in the decomposition processes, where derived kinetic scheme must be compatible in kinetic sense and thus actual kinetic scheme must possess high accuracy and reliability in modeling of pyrolysis.

Using the actual forms of *E*–α dependencies (Fig 6), it can be concluded that such trends of the variation of *E* values with conversion are typical for lignocellulosic biomass pyrolysis [47]. This behavior can be associated with overlapped decomposition reactions of hemicelluloses, cellulose, and lignin. Therefore, the application of DAEM includes particularly the decomposition of every pseudo-component.

### 4.4. Modeling the pyrolysis processes

**Fig 7:**
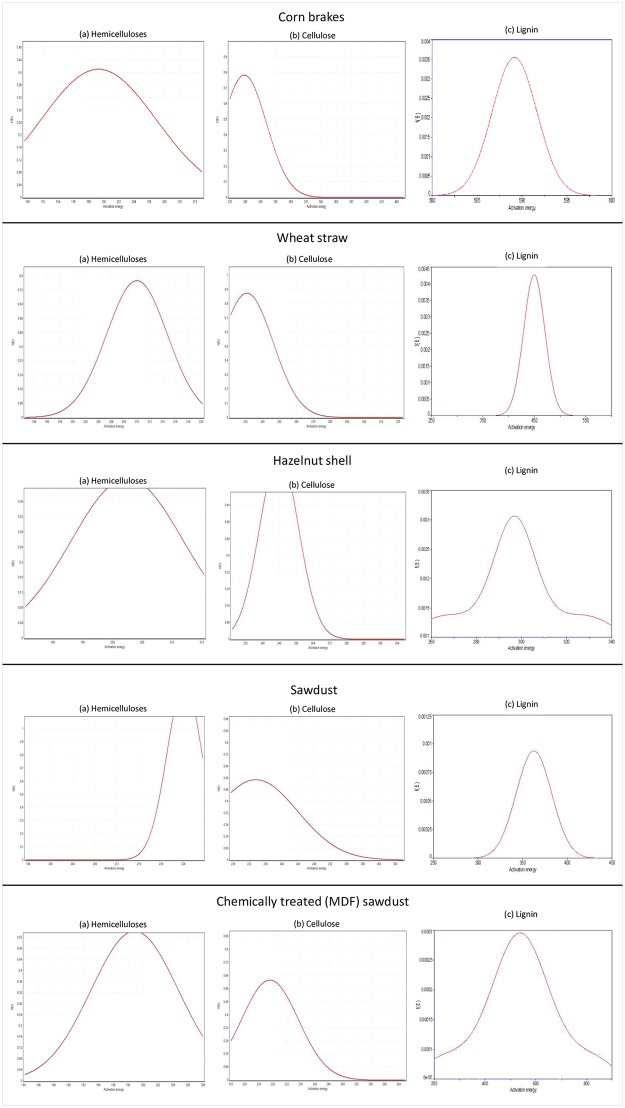
The Gaussian distribution curves of activation energies for hemicelluloses, cellulose and lignin decompositions.

The experimental results have implemented in the process of obtaining the *n*-th order Gaussian (Normal) DAEM prediction for all investigated pyrolysis processes. After the process of estimating the initial distribution curves within parallel three-step pyrolysis process including hemicelluloses, cellulose and lignin decompositions, corresponding probability density distributions of activation energies were estimated. The likelihood of occurrences and the shapes of a given distributions obtained for actual kinetics are shown in Fig 7 ((a)–hemicelluloses; (b)–cellulose; (c)–lignin).

It can be observed that within the same class of biomass samples (the agricultural by-products), the largest spread in the distribution of activation energies shows corn brakes and hazelnut shell samples for hemicelluloses decomposition, while in the case of wood by-products, such behavior is noted for chemically treated sawdust (Fig 7). All distributions are characterized with bell-shaped forms. For cellulose decomposition, the bell-shaped form of distributions still exists but the distributions show some asymmetry that is not typical for Gaussian type of distribution functions. The greatest tendency towards symmetry between these distributions shows the distribution for the decomposition of cellulose in the case of hazelnut shell pyrolysis (Fig 7). However, among these distributions, the main difference can be underlined on the basis of their appearance, where there are certain differences in those present in the case of agricultural by-products and in the case of wood by-products. These differences will also cause changes in the kinetic parameters of the decomposition process of individual pseudo-components. On the other hand, in the case of lignin distributions, we have an interesting situation. Two activation energy distributions are distinguished by their form from all others. Namely, for hazelnut shell and MDF sawdust pyrolysis processes, the lignin decomposition was characterized by “winged” Gaussian distributions, where for hazelnut shell this is quite pronounced in such a form, that the tails create a “raised wings”. This phenomenon was not observed in other samples. Thus, the identified behavior of the distribution of reactivity is related to a specific form of *E* = *E*(α) dependency estimated for hazelnut shell pyrolysis (Fig 6). Raising the “wings” for lignin distribution of reactivity attached to hazelnut shell and MDF sawdust pyrolyses (Fig 7) is a consequence of the presence of multiple numbers of reactions, which are obviously different from the number of reactions that take place in other biomass systems, during their decomposition pathways. Therefore, this behavior must be treated as existence of dichotomy between lumped kinetic model and distributed activation energy mode. This is reflects in the number of expected decomposition reactions. In observed cases, the mathematical model is obviously unable to simulate more than one maximum but in reality, there are three “hidden” maximums. Therefore, the Gaussian distributions with appearance of “wings” for lignin decomposition reactions in the case of hazelnut shell and MDF sawdust pyrolysis processes, apparently looks more suitable for materials, which comprise of many different volatile components.

From the simulation procedure, the generated data were established. The results are shown in Table 3. Results presented in Table 3 are related to three-step kinetic scheme consisting of decompositions of the main pseudo-components of biomass.

**Table 3:** The *n*-th order model with Gaussian distribution functions as well as kinetic parameters related to hemicelluloses, cellulose and lignin decompositions for various biomass samples; parameters belong to the kinetic scheme of three-parallel independent reactions; dispersion values (*σ*) in given table do not refer to the deviation caused by the “wings” phenomenon appeared in Fig 7 for lignin decomposition within Hazelnut shell and MDS pyrolysis processes.

Sample	Corn brakes			Wheat straw			Hazelnut shell			Sawdust (Beech)			Chemically treated (MDF) sawdust		
PC ^a^	1 ^b^	2 ^c^	3 ^d^	1 ^b^	2 ^c^	3 ^d^	1 ^b^	2 ^c^	3 ^d^	1 ^b^	2 ^c^	3 ^d^	1 ^b^	2 ^c^	3 ^d^
Parameters															
***μ*** **(kJ mol**^**-1**^**)**	199.31	238.73	589.16	209.98	230.83	449.65	202.47	247.84	296.85	224.16	233.58	362.53	198.84	227.17	538.83
***σ*** **(kJ mol**^**-1**^**)**	7.580	29.224	2.492	4.747	15.406	20.611	9.474	9.181	8.711	3.140	10.316	19.905	5.843	8.103	113.693
***n*** ^e^	3.50	5.22	2.35	2.75	3.51	1.98	1.85	2.65	2.41	4.47	1.53	1.36	8.60	2.45	1.40
***A* (min**^**-1**^**)**	1.6729 × 10^29^	5.0709 × 10^35^	6.3751 × 10^41^	2.1842 × 10^19^	7.9579 × 10^24^	8.012 × 10^38^	5.4018 × 10^22^	1.3141 × 10^45^	3.4481 × 10^24^	3.2217 × 10^18^	4.4450 × 10^20^	2.9260 × 10^26^	5.1805 × 10^17^	1.4921 × 10^20^	2.8910 × 10^40^
***w*** ^f^	0.29	0.40	0.31	0.30	0.44	0.26	0.16	0.23	0.61	0.31	0.45	0.24	0.29	0.49	0.22

^**a**^ Pseudo-component.

^**b**^ Hemicelluloses.

^**c**^ Cellulose.

^**d**^ Lignin.

^**e**^ Apparent reaction order.

^**f**^ Weight parameter.

Taking into account all pseudo-components in biomasses, the mean activation energy values are in the range of 198.84–589.16 kJ mol^-1^, and by varying the apparent reaction orders in the range of 1.36–8.60 (Table 3). It can be observed that the kinetics od pseudo-components decomposition deviates from the simple first-order reaction model, and obeys to the more complicated reaction mechanisms with *n* ≠ 1 (this is not unusual behavior given the complex chemical structure of biomass, and the large variety of chemical linkages). The great dissipation in *n* values is a consequence of the large variety of the decomposition reaction pathways goes from one to another biomass sample. This is specifically pronounced if we take into account different categories of biomass feedstocks.

For wood by-products pyrolysis, the decomposition of hemicelluloses was governed by a very high values of apparent reaction order (*n* > 4.00), while the high apparent reaction order was identified for cellulose decomposition for corn brakes pyrolysis (*n* = 5.22) (Table 3). Within the same category of biomass feedstock, it can be observed that the lignin decomposition was characterized with lower *n* values, but with high mean activation energy values. For agricultural by-products, the *n* value is somewhat higher, but there is a certain decrease in the mean activation energies for lignin decomposition (the exception is only corn brake sample) (Table 3). The activation energies of the lignin extend through a high *E* interval, which demonstrates that lignin has a much complex structure than the other constituents and has an extremely wide activity range for chemical bonds and functional groups. The distribution of the pre-exponential factors has a closely related behavior to the distribution of the activation energies for all observed cases. In Table 3, the best optimized values of the pre-exponential factors were presented for all investigated samples. From dispersion (standard deviation—*σ*) values, we can obtain the additional information about the reactivity of given pseudo-components in given samples during their pyrolysis process.

For agricultural by-products, the dispersion (*σ*) values follow the order such as: CB: *σ*_C_ > WS: *σ*_L_ > HS: *σ*_H_ (where C, L, and H designates the cellulose, lignin, and hemicelluloses, respectively) (Table 3). This indicates that the pyrolysis of these pseudo-components in observed samples occurs over an extensive temperature range. The larger the value of *σ*, the broader is the reaction profile. On the other hand, for wood by-products, the following order of dispersion (*σ*) values was established: CTSD (MDF): *σ*_L_ > SD: *σ*_L_ (Table 3). For both samples, the third pseudo-component (lignin) shows a significant reactivity that is distinguished by the distribution functions of activation energies, especially for CTSD (MDF) sample (Fig 7).

**Fig 8:**
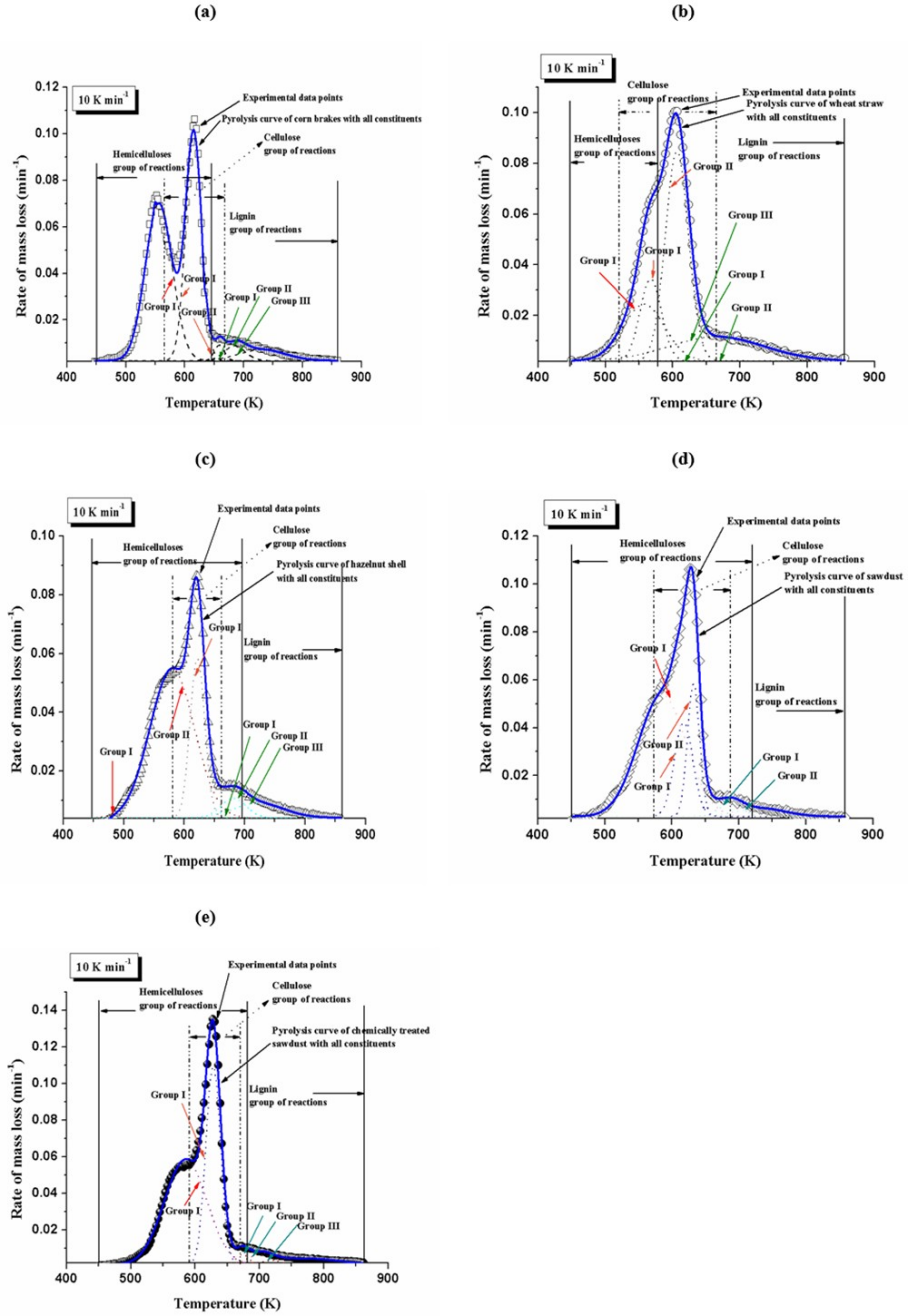
Pyrolysis process of various biomasses under the heating rate of 10 K min^-1^, including the decomposition reactions of the constituents representing the entire pyrolysis behavior; comparison between experimental and calculated three-parallel independent reaction rate curves is also presented.

According to the kinetic parameters derived, the experimental curves of mass loss rate were reconstructed, which are characterized with dominating group of reactions under each pseudo-component decomposition process. Fig 8 shows the pyrolysis process and characteristics contributed from different constituents for all investigated biomasses, at the heating rate of 10 K min^-1^.

It can be observed from Fig 8 that regarding the biomass type, every pseudo-component forms a characteristic group of reactions at certain temperature intervals. Taking into consideration CB, WS and HS (Fig 8a–8c), each constituent decomposes in different stages for difference of physico-chemical structures. The more complex the structure, the more difficult the decomposition becomes. Namely, for CB and WS, hemicelluloses form only one group of reactions (Group I), while for HS, the same pseudo-component forms two groups of reactions (Groups I and II). In the case of cellulose decomposition, for HS its forms only one group of reactions, while for CB and WS, the cellulose forms two groups of reactions. For all systems, the lignin forms three groups of reactions (Group I, II, and III) (Fig 8a–8c). From actual observation, the hemicellulose is thermally most sensitive and is the first one beginning to decompose as the increase of temperatures. However, depending on the observed sample and the percentage participation of hemicelluloses in these samples, the temperature range of the hemicelluloses decomposition varies. For CB and WS, the decomposition of hemicelluloses mainly takes place around 550 K, while for HS, it starts at a slightly higher temperature and reaches the maximum around 575 K. With increasing the further the temperature, for all samples, the decomposition of the hemicelluloses almost completed at 650 K. Concurrently, around the temperature of 575 K, the cellulose starts to decomposes and reaches the maximum rate of mass loss around 610 K. However, the starting and ending decomposition temperatures of cellulose vary by referring these biomass samples (Fig 8a–8c). Finally, the lignin has a broad decomposition region separated with three main groups of reactions in distinctive temperature intervals. However, broadness of its reactivity significantly varies from one to another sample (Fig 8a–8c). During the pyrolysis processes, it is obvious that the decomposition regions of the constituents overlap each other, which clearly demonstrates there are complex interactions among the constituents.

In the case of SD and CTSD (MDF), we have a different situation than previously described. Namely, for SD sample, hemicelluloses first begin to decompose but this process is followed by strong overlapping with two groups of reactions attached to cellulose decomposition (Fig 8d). This is in full agreement with the mean activation energy values for the decomposition of hemicelluloses and celluloses presented in Table 3, since these values are very close. On the other hand, for CTSD (MDF) sample, this complexity is not so pronounced where it can be seen that the decomposition of cellulose in this case takes place through only one group of reactions. This also can be identified by decreasing of *μ* values for cellulose (Table 3). In the case of lignin, its decomposition process runs through a variety of group reactions (two groups of reactions (Group I and II) for SD, and three groups of reactions (Group I, II and III) for CTSD (MDF)) (Fig 8d and 8e). The increase in reaction groups is accompanied by an increase in the *μ* value for lignin decomposition in the case of wood by-products pyrolysis.

Regarding to evolution of the rate of mass loss with temperature compared to the experimental results (Fig 8), the model produces very good data. The results calculated by the model distinguish the influence of cellulose and hemicelluloses with a first knee at lower temperatures (related to hemicelluloses) than the main peak (related to cellulose) for HS among agricultural by-products. This fact could be explained that for HS pyrolysis, the quite weak interaction between constituents exists, while a much stronger interaction exists in the case of SD pyrolysis, among wood by-products. Therefore, this additive model of principal constituents can successfully describe the pyrolysis process both numerically and analytically, considering totally different types of biomass.

Therefore, Table 4 summarizes all the results shown in Fig 8 related to the group of reactions taking place during the decomposition of hemicelluloses (H), cellulose (C) and lignin (L).

**Table 4:** Summary of the group of reactions taking place during the decomposition of hemicelluloses (H), cellulose (C) and lignin (L).

Sample	Corn brakes			Wheat straw			Hazelnut shell			Sawdust (*Beech*)			Chemically treated sawdust		
*T* (K)	Reaction group														
	H ^a^	C ^b^	L ^c^	H ^a^	C ^b^	L ^c^	H ^a^	C ^b^	L ^c^	H ^a^	C ^b^	L ^c^	H ^a^	C ^b^	L ^c^
**485, 550, 570** ^d^	Cracking and abscission of C-C and C-O bonds	-	-	Cracking and abscission of C-C and C-O bonds; Cracking and reforming of C = O group	-	-	Distinguished contribution of pentosans that are highly susceptible to hydrolysis and dehydration reactions; especially at 585 K the cracking of C = O groups dominates	-	-	Higher than 570 K, the interaction with cellulose may causes reducing in cellulose stability; C-O-C bond rupture, alongside with pyranose C-C bond rupturing	-	-	Cracking and abscission of C-C and C-O bonds; the presence of impurities may causes that a less tar can be fomed during this stage of the process	-	-
**610, 630, 660** ^d^	-	Cracking of carboxyl (C = O) and carbonyl (C-O-C) groups	-	-	Cracking of carboxyl (C = O) and carbonyl (C-O-C) groups	-	-	Cracking of carboxyl (C = O) and carbonyl (C-O-C) groups	-	-	Levoglucosan formation from intermediate radicals, and increase of carbonyl groups	-	-	The presence of organic preservative probably affects the levoglucosan formation and on the overall cellulose decomposition	-
**630, 665** ^d^	-	-	Disrupting of O-CH_3_ bond contents	-	-	Disrupting of O-CH_3_ bond contents	-	-	-	-	-	-	-	-	Disrupting of O-CH_3_ bond contents
**670, 690** ^d^	-	-	Primary releasing of volatiles such as H_2_ and CH_4_, because of higher content of aromatic ring and O-CH_3_ groups	-	-	Primary releasing of volatiles such as H_2_ and CH_4_, because of higher content of aromatic ring and O-CH_3_ groups	-	-	Primary releasing of volatiles such as H_2_ and CH_4_, because of higher content of aromatic ring and O-CH_3_ groups	-	-	Primary releasing of volatiles such as H_2_ and CH_4_, because of higher content of aromatic ring and O-CH_3_ groups	-	-	Primary releasing of volatiles such as H_2_ and CH_4_, because of higher content of aromatic ring and O-CH_3_ groups
**720, 750** ^d^	-	-	Sternly cracking and deformation of lignin structure; significant contribution to charring process	-	-	-	-	-	Sternly cracking and deformation of lignin structure; significant contribution to charring process	-	-	Sternly cracking and deformation of lignin structure; significant contribution to charring process	-	-	Sternly cracking and deformation of lignin structure; significant contribution to charring process

^**a**^ Hemicelluloses.

^**b**^ Cellulose.

^**c**^ Lignin.

^**d**^ Difference might be due to the inherent variance among the chemical structure of the three pseudo-components such as hemicelluloses appeared more C = O contained organics compounds, while higher contents of -OH and C–O can be found with celluloses.

Depending on the type of biomass, the processes vary at certain temperature intervals, which depend to a large extent on the chemical structure of biomass constituents. However, based on the obtained kinetics parameters, resolving of the main pyrolysis reactions was performed, which are very close to the published data in the literature [43].

**Fig 9:**
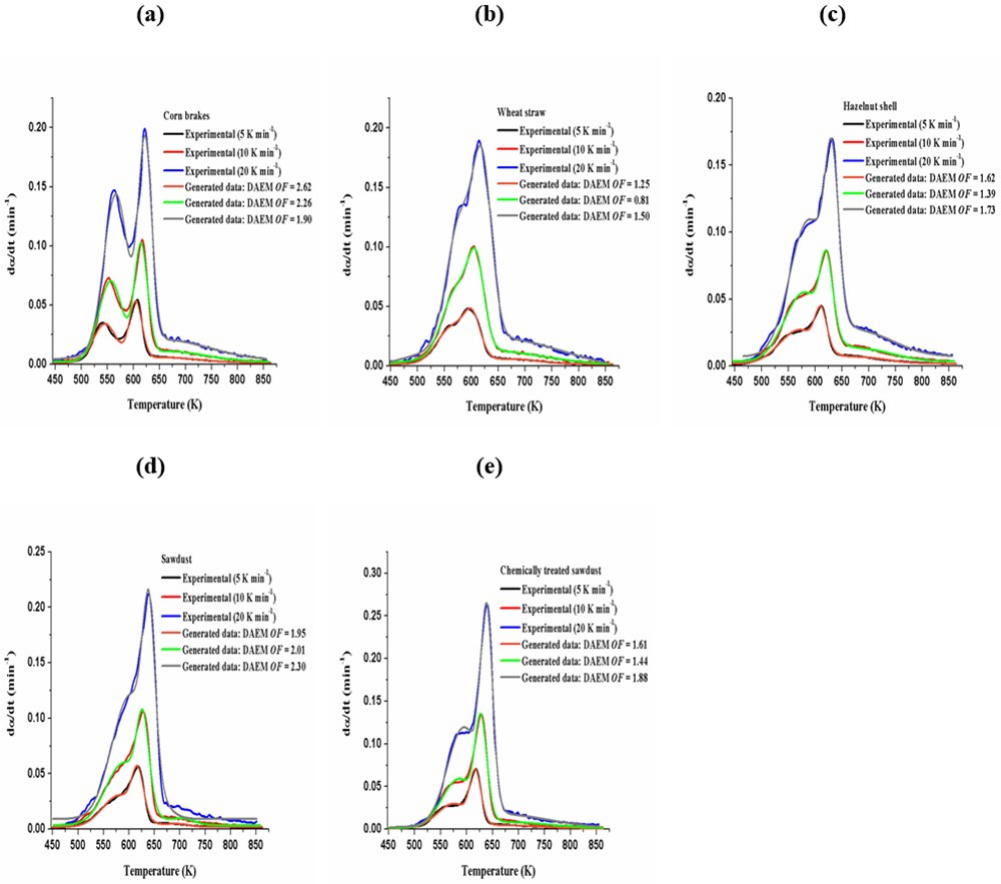
Experimental pyrolysis rate (absolute) curves at different heating rates and calculated curves fitted with three-parallel independent reaction model, for (a) corn brakes, (b) wheat straw, (c) hazelnut shell, (d) sawdust, and (e) chemically treated (MDF) sawdust.

Fig 9a–9e shows the comparison of the pyrolysis rate (absolute values) curves deduced experimentally and those obtained from the three-parallel independent reaction model (hemicelluloses + cellulose + lignin (Table 3)), without emphasized groups of decomposition reactions, related to particular pseudo-component. In the calculation procedure, the Eq (7) was used.

It can be observed from Fig 9 that superimposition of experimental and fitted curves exists at all heating rates. A good agreement was obtained between experimental and modeling pyrolysis rate curves with *OF* less than 3.20% for applied model. However, for corn brakes at the lowest heating rate, and for sawdust at the highest heating rate, some disagreement can be noticed. These samples belong to the different class of biomass feedstock and react differently with a change in the heating rate, but the significant impact of thermal lag can appear for the higher heating rates. However, the general problem with simulation of thermal decomposition of materials that comprises various other compounds, as number of the maximums, is independent of behavior of the individual pseudo-components with time. Nevertheless, the proposed three-parallel independent reaction model places a higher maximum for reaction rate in the vicinity of the temperature to the experimental ones, so actual model based on three main pseudo-components of studied biomasses produces a very good approximation of pyrolysis process. Also, the actual model provides the possibility of application for the multiple distributions, as when different compounds with different mean and dispersion values can be combined.

## Conclusions

In this work, the pyrolysis process of various types of biomasses (including agricultural and wood by-products) in non-isothermal conditions was investigated. The pyrolysis processes were monitored on the laboratory scale-grades using the simultaneous thermal analyses (STA).

The devolatilization kinetics was implemented through combined application of model-free methods and DAEM (distributed activation energy model) using Gaussian distribution functions of activation energies (*E*). It was found that three-parallel independent reaction model proposed in this work is more suitable than two-parallel reaction model for the kinetic description of the complex kinetics of lignocellulosic biomass pyrolysis and might also be used to estimate the composition of different lignocellulosic biomass feedstocks using the thermal techniques.

The good comparison of experimental and calculated data allows the use of proposed model to generate also the good approximations of the temperature ranges where the reaction will take place within the slow pyrolysis conditions, where pyrolysis would be considered as pretreatment for gasification or combustion processes. The actual model also describes the influence of kinetic parameters and activation energy distribution characteristics on the behavior of the pseudo-component decomposition reactions. As a result of application of the numerical optimization through comparison of predictions and the experimental data, the proposed model may be used to produce a very good approximation of pyrolysis process for all studied biomass samples including their major constituents, from the variation of the rate of mass loss with temperature. Also, the actual model can help in optimizing the pyrolysis process of different biomass pseudo-components (hemicelluloses, cellulose, and lignin) and can be used as a tool to control working conditions in future integrated biomass feedstock systems.
